# Systematic review of economic evaluation of early-life nutrition interventions to promote healthy eating during the first 1000 days

**DOI:** 10.1017/S1368980026103061

**Published:** 2026-07-13

**Authors:** Kim Jeong, John Cairns

**Affiliations:** 1 Institutt for ernæring og folkehelse, University of Agder, Norway; 2 London School of Hygiene & Tropical Medicine, UK

**Keywords:** Cost-effectiveness, Economic evaluation, Nutrition, Healthy eating, First 1000 days

## Abstract

**Objective:**

To identify economic evidence and evaluation for early-life interventions promoting healthy eating in the first 1000 days and to establish evidence gaps.

**Design:**

Five databases and five websites were searched using key words. Findings were synthesised narratively.

**Setting:**

Home, education and community settings.

**Participants:**

Pregnant women, infants and young children aged 0–2 years.

**Results:**

Twenty-four English-language, peer-reviewed economic evaluations reporting costs and consequences were selected. Overall, included studies demonstrated mixed quality. Using the Consolidated Health Economic Evaluation Reporting Standard (CHEERS) 2022, titles, comparators and outcomes were commonly reported, whereas health economic analysis plan (HEAP), heterogeneity and uncertainty were least reported. Four studies stated the analytical perspectives with clear decision contexts, making the results useful in decision-making. Several studies had a time horizon of one year or shorter with unclear decision context. Four studies presented relatively transparent methods, with the interpretation of results from cost–utility analyses being informative for decision-making.

**Conclusions:**

This review identified consistent methodological challenges in the economic evaluation of early-life nutrition interventions. Comparators were inconsistently defined, and most studies adopted short time horizons, limiting assessment of longer-term outcomes. Chosen outcomes were focused on immediate health outcomes, with limited consideration of broader impacts or population differences. More robust and transparent approaches to economic evaluations, supported by a HEAP could improve consistency and support evidence-based decision-making.

Promoting healthy eating behaviours in the first 1000 days is important for long-term health. Healthy eating behaviours may influence the risk of developing non-communicable diseases (NCD), including obesity, type 2 diabetes, cardiovascular diseases and depression^(1)^. Therefore, it is important to understand the cost-effectiveness of interventions aimed at improving healthy eating behaviours. Healthy eating is defined as eating a variety of whole (i.e. unprocessed) and fresh food every day and avoiding food high in sugar, fats and salt^(2)^. The term ‘first 1000 days’ refers to a period from conception to a child’s second birthday during which rapid growth and development take place and where the foundations for future health and wellbeing are built, making optimised nutrition including healthy eating behaviours critical for healthy development for the newborn^(3)^. Parents’ and primary carers’ prior knowledge, behaviours and beliefs on healthy eating have lasting impacts on the formation of eating habits of newborns and infants. Therefore, early-life nutrition interventions to promote healthy eating during the first 1000 days are critical for achieving health and wellbeing benefits related to meeting nutritional needs and by preventing overweight, obesity and NCD. The importance of economic evaluation for cost-effective resource allocation to improve child nutrition in low- and middle-income countries was highlighted in a recent systematic review^(4)^. However, a review of economic evidence related to nutrition interventions to promote healthy eating in the first 1000 days is lacking.

This review aims to identify economic evidence and methodological approaches used in evaluations of early-life nutrition interventions to promote healthy eating in the first 1000 days in community, education and home settings and establish evidence gaps. Evidence gaps and challenges identified from this review will highlight areas for future research.

## Key messages

- Economic evaluation of early-life nutrition interventions to promote healthy eating during first 1000 days are typically embedded within complex public health interventions, making it challenging to identify relevant comparators and to capture costs and consequences.
- Health service utilisation, earnings, strengthened workforce, reduction in NCD and health-related quality of life (HRQoL) were considered as important outcomes but not reported in the included studies.
- A health economic analysis plan (HEAP) should be an integral part of a study protocol, including defined economic questions, population, intervention, comparator(s), outcomes (PICO), analytical perspective(s), the approach to handling uncertainties and decision contexts.
- Pathway modelling along the early child development (ECD) stages provides an opportunity to capture both costs and the full range of intervention impacts.

## Methods

### Search strategy

**Figure 1.**
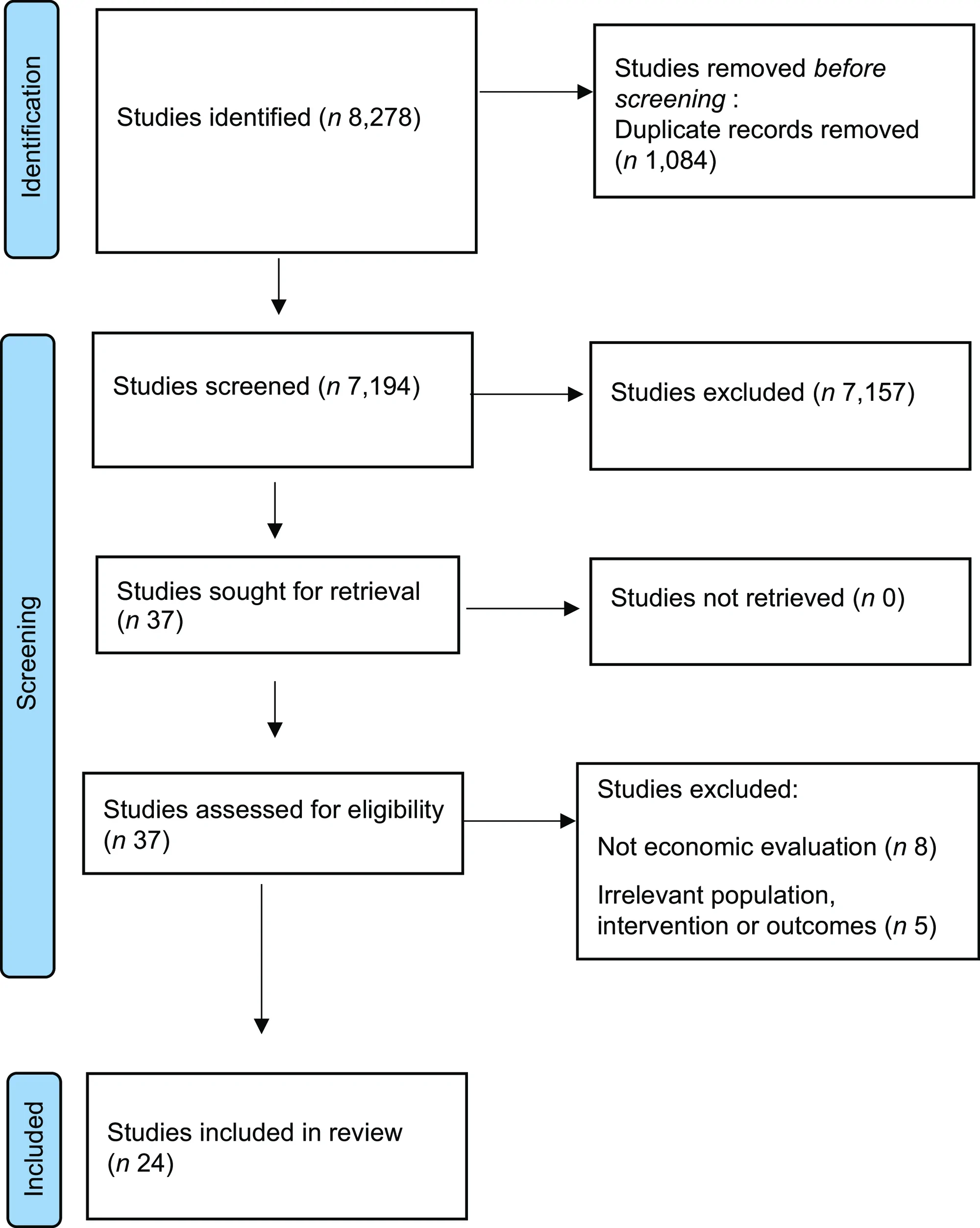
Preferred Reporting Items for Systematic reviews and Meta-Analyses (PRISMA).

A search strategy was developed using cost-effectiveness, economic evaluation, nutrition, healthy eating, first 1000 days, children in home, education and/or community settings as key words (Appendix 1). Geographic search filters were not used to ensure capturing relevant studies. Databases searched include Embase, MEDLINE, EconLit and HTA database. WHO websites, Google (restricted to the UK domains) and Google Scholar were searched, and, where appropriate, the first fifty results were examined to identify any relevant publications that have not already been identified through database searches. From identified literature, duplicates were removed, and the titles and abstracts were screened based on eligibility criteria (Appendix 1). The literature search was conducted on 11 April 2024 and identified 7194 studies after removing duplicates. After screening titles and abstracts based on eligibility criteria, thirty-seven studies were selected for full-text review. The Preferred Reporting Items for Systematic Reviews and Meta-Analyses (PRISMA) is shown in Figure 1.

Studies were included based on the following criteria:

- Population: pregnant women, infants and young children aged 0–2 years in home, education and community settings.
- Intervention: activities such as education, feeding or dietary practices to promote healthy eating. Healthy eating includes breast-feeding, daily consumption of grains, protein, dairy products, fruits and vegetables while limiting intake of processed foods, saturated and trans fats, salt and sugar.
- Comparison: usual or standard practice, including do nothing, where appropriate.
- Primary outcomes – improved frequency of healthy eating (e.g. changes in the weekly/monthly fruits and/or vegetables intake, starting and continuing breast-feeding, BMI, BMI-*z* score, height, weight and existing anthropometric measures.
- Secondary outcomes – HRQoL, quality-adjusted life year (QALY) and disability-adjusted life year (DALY).
- Study design: cost–utility analysis (CUA), cost–benefit analysis (CBA), cost-effectiveness analysis (CEA), cost–consequence analysis (CCA) and DALY averted.
- Publication types and years: peer-reviewed publications in English language from 1 January 2000 to April 2024.

Studies were excluded based on the following criteria:

- Title/abstract that are irrelevant to nutrition interventions for the first 1000 days.
- Conference abstracts or study protocols with no full publication of economic evaluation.
- Unspecified or not clearly defined early-life nutrition interventions.
- Nutrition interventions for clinical diagnosis.
- Interventions for children older than 2 years or non-pregnant women.
- Interventions related to clinical diagnosis, food (in)security, malnutrition, food fortification, micronutrients and home parenteral/enteral feeding.

### Study selection and quality assessment

The full text of selected studies was assessed independently by both reviewers. The Consolidated Health Economic Evaluation Reporting Standards (CHEERS 2022) checklist was used to assess reporting quality^(5)^. Methodological quality was assessed using a widely recognised framework for evaluating economic evaluations, including study design, well-defined decision problem, identifying comparator(s) and perspective on costs and consequences, time horizon and discounting, synthesis on health effects, evidence on resource use and costs and the chosen modelling approach to measuring outcomes, sources of data for measurement of HRQoL^(6)^ such as European Quality of Life 5 Dimensions (EQ-5D), Short-Form 6-Dimensions (SF-6D), 15-dimensional HRQoL (15D), source of preference data for valuation of changes in HRQoL, handling uncertainties in costs and consequences, interpretation of cost-effectiveness results and the interpretation of results compared to those of other studies. Any disagreements were resolved through discussion at each stage. EndNote® and Microsoft Excel® were used for the data management. Please see the PRISMA (Figure 1). Search results are in Appendix 2. Findings are presented in the following section, grouped by intervention aims to facilitate comparison, and a narrative synthesis focusing on methodological approaches was undertaken due to substantial heterogeneity.

## Results

**Figure 2.**
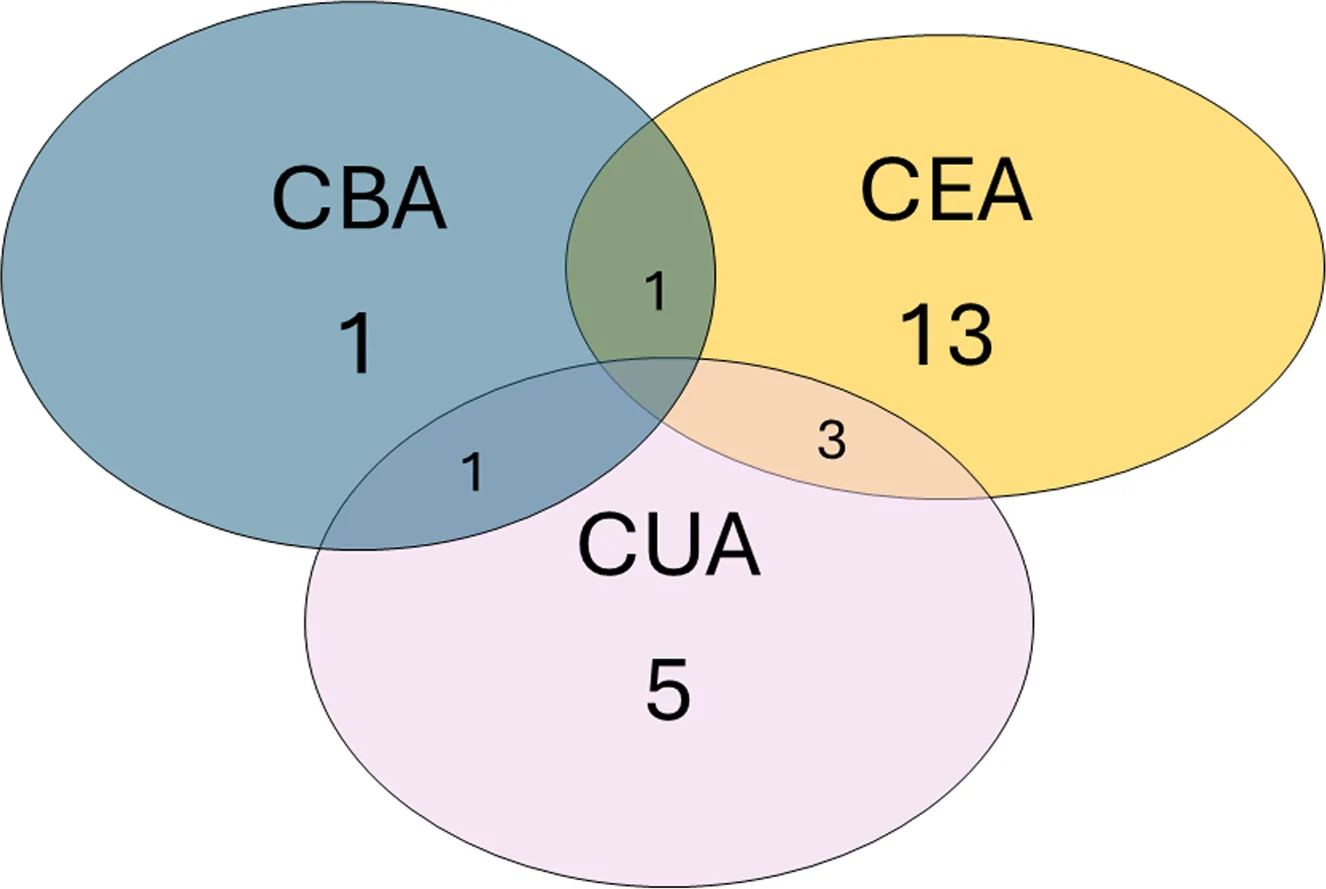
Included studies by study types. CBA, cost–benefit analysis; CEA, cost-effectiveness analysis; CUA, cost–utility analysis.

The literature search was conducted on 11 April 2024 and identified 7194 studies after removing duplicates. After screening titles and abstracts based on eligibility criteria, thirty-seven studies were selected for full-text review (Figure 1). Of thirty-seven studies, thirteen studies were excluded after independent reviews and discussions by two reviewers (Appendix 3). The common reasons for exclusion were that studies were not economic evaluations or that the study population, intervention or reported outcomes were irrelevant. Of twenty-four included studies, the most common type of economic evaluation was CEA (Figure 2). The studies varied by setting; one multi-centre European randomised controlled trial (RCT)^(7)^, four studies were in Australia^(8–11)^, five in the UK^(12–16)^, two in Finland^(17,18)^, two in the USA^(19,20)^, two in Uganda^(21,22)^ and one in Guatemala^(23)^, Burundi & Guatemala^(24)^, South Africa^(25)^, Pakistan^(26)^, Peru^(27)^, Kenya^(28)^, Sweden^(29)^ and Vietnam^(30)^ (Table 1).

**Table 1:** Summary of included studies Table 1 long description.

Study (year)/setting	Population	Intervention	Comparator(s)	Outcomes	Type of economic evaluation	Viewpoint(s)	Time horizon/discounting
Bailey (2022)/Australia	Singleton pregnancies with a birth of more than 20 weeks’ gestation	Dietary advice and physical activity	Standard care	Cost per case (gestational diabetes mellitus (GDM), hypertensive disorder in pregnancy or both) prevented, cost per birth type (vaginal, caesarean, induction)	CEA	Public healthcare system	Less than 1 year (during pregnancy)/not applicable (NA)
Broekhuizen (2018)/9 EU countries	Pregnant women with a pre-pregnancy BMI of ≥ 29 kg/m^2^	Dietary advice (healthy eating) and physical activity	Standard care	Gestational weight gain, fasting glucose, insulin resistance (HOMA-IR), quality-adjusted life years (QALY)	CUA	Societal	Less than 1 year (during pregnancy)/NA
Dodd (2015)/Australia	2213 pregnant women who are overweight and obese	Dietary advice, physical activity, and behavioural strategy	Standard care	Incremental cost-effectiveness ratio (ICER) in preventing one additional infant with birth weight above 4 kg, ICER in preventing one additional infant with moderate to severe respiratory distress syndrome	CEA	Healthcare system	Less than 1 year (pregnancy-6 weeks post-partum)/NA
Kolu (2013)/Finland	Pregnant women with risks of GDM	Intensified counselling on physical activity, diet and appropriate weight gain	Standard care (no extra counselling)	ICER for antenatal service, quality of life using 15D and visual analogue scale (VAS)	CEA, CUA	Societal	9 months (during pregnancy)/NA
Kolu (2016)/Finland	Participants of Kolu study (2013) and their children who were born during the intervention, a 7-year follow-up study	Intensified counselling on physical activity, diet and appropriate weight gain	Standard care (no extra counselling)	ICER for antenatal service, quality of life using 15D and VAS	CEA, CUA	Societal	7 years and lifetime/no discounting, but QALY gain is based on a lifetime horizon
Poston (2017)/UK	Pregnant women with a BMI of ≥ 30 kg/m^2^ and a 15^+0^ to 18^+6^ weeks’ gestation	Diet, physical activity, behavioural intervention	Standard antenatal care	GDM, large-for-gestational-age delivery	CUA	NHS acute care provider	Less than 1 year/NA
Rogozinska (2017)/UK	Pregnant women with no predefined disorders	Diet and physical activity	Standard care	Cost per case of pre-eclampsia, pregnant-induced hypertension, GDM, any major complications avoided	CEA	NHS	Less than 1 year/NA
Nianogo (2019)/USA	Hypothetical cohort of 500 000 Californian pregnant women	Pregnancy, prenatal Women, Infants and Children (WIC) coverage: status quo (84 %), universal coverage (100 %)	Absence of WIC (0 % coverage rate)	Number of preterm birth cases prevented, benefit:cost ratio	CBA, CUA	Societal	Newborn’s life course/no for intervention costs (within the year), 3 % for future cost savings and QALY gained
Jacklin (2007)/UK	Lactating women and infants	Ranges of breast-feeding initiation – hypothetical scenario analysis	What-if analyses; compares a peer support scheme to no peer support scheme (neither very clearly described)	Threshold analyses to identify the ranges in which the intervention would be cost-effective	CUA	Health service provider, daily cost of formula milk	Lifetime/no discount on costs, 3·5 % on QALY gained
Walters (2016)/Vietnam	Women with infants, young children feeding	Breast-feeding – a comprehensive breast-feeding strategy in Vietnam	No national strategy breast-feeding	Benefit:cost ratio of modelled breast-feeding strategy	CBA	Provider, some costs borne by household	1 year/NA comparison of costs and benefits in a single year
Anokye (2020)/UK	5398 mother–infant dyads (intervention), 4612 mother–infant dyads (control)	Financial incentives (vouchers) for breast-feeding	No offer of financial incentives	Babies breastfed (receiving breast milk) at 6–8 weeks, and cost per additional baby breast fed	CUA	Provider	Within 1 year/NA given time horizon and outcomes used
Chola (2015)/Uganda	329 mother–infant pairs (intervention) and 368 (control group) from birth to 24 weeks	Peer counselling plus health facility breast-feeding promotion	Health facility breast-feeding promotion	Cost per (additional) month of exclusive or predominant breast-feeding, cost per DALY averted	CEA, CUA	Provider	6 months/3 % for DALY
Hoddinott (2012)/UK	Women admitted to postnatal ward after birth	Proactive telephone support for breast-feeding promotion	Reactive telephone support	Incremental cost of providing proactive calls per additional woman breast-feeding and per additional woman exclusively breast-feeding at 6–8 weeks	CEA	Healthcare provider (not explicit)	6–8 weeks/NA
Pugh (2002)/USA	41 low-income women postpartum	Breast-feeding-promotion strategy with supplementary support for breast-feeding	Usual breast-feeding support	Breast-feeding time	CEA	Implicitly societal (included some costs to mothers, health service costs)	6 months/NA only a 6-month horizon
Wynn (2017)/South Africa	645 pregnant women (18 years and older) had less than 34 week’s gestation (intervention) and 594 (standard care group)	Home visits for breast-feeding	Standard care	ICER for low birth weight (LBW) infant averted, stunting averted and exclusive breast-feeding added (note they share the same incremental costs)	CEA	Provider	2 years/6 % discount rate to annuities certain costs
Hayes (2014)/Australia	667 first-time mothers and their infants	Home visits and counselling for early childhood obesity prevention	Standard care	ICER per unit BMI avoided and per 0·1 reduction in BMI-*z* score	CEA	Health funders	2 years
Doring (2018)/Sweden	1355 families with 1369 infants	Motivational interviews promoting healthy food and physical activity habits	Standard care	BMI at age 4	CEA	Societal	3 years/no discounting
Perry (2023)/Guatemala	Pregnant women or mother of an under-2 child	Routine and systematic home visits	Not stated	Cost per DALY averted	CUA	Not stated	Not stated/not stated
Heckert (2020)/Burundi, Guatemala	Mother–child pairs	Nutrition education – Behaviour change communication (BCC) + food assistance/rationing for maternal and child health	Business as usual (Guatemala); no food ration during pregnancy (Burundi)	Cost per beneficiary per percentage point reduction in stunting	CEA	Provider	2 years/3 % (to annuities start-up costs)
Waters (2006)/Peru	187 children (intervention) and 190 (control) from birth to 18 months	Infant nutrition education	Standard care (control)	Cost per stunting case averted	CEA	Provider plus some household costs	1 year/no discounting
Gowani (2014)/Pakistan	Children from birth to 24 months	Nutrition education – Enhanced Nutrition (EN), responsive stimulation (RS), EN combined with RS	No responsive stimulation, no enhanced nutrition	Bayley Scale for Infant and Toddler Development (BSID III)	CEA	Public sector	2 years/no discounting
Ahmed (2023)/Uganda	Total 511 mother–child dyads, intervention (*n* 263) and control (*n* 248) at baseline	Maternal nutrition education	Standard care	Cognitive composite score gained (BSID-III)	CEA	Healthcare provider	18 months/no discounting
Garcia (2021)/Kenya	376 mother–child dyads (group only), 400 (mixed delivery), 376 (comparison)	Group sessions with incentivised attendance, (soap/milk) with home visits with repeated messages on child feeding – parenting programme for early childhood development (ECD)	Standard care	Additional sd per USD100	CEA, CBA	Provider, societal	1 year (within the trial); lifetime (CBA)/5 % CBA
Reeves (2021)/Australia	0–6 years of children in 54 childcare service centres	Web-based tool for menu planning to improve implementation of dietary guidelines	Standard care	Relative value index (RVI), calculated by dividing the usual practice average cost-effectiveness ration (ACER) by the ICER calculated for the new intervention	CEA	Healthcare, modified societal (costs to childcare services)	1 year/no discounting

CEA, cost-effectiveness analysis; CUA, cost–utility analysis; NHS, National Health Service; DALY, disability-adjusted life year.

Comparator terminology varied across studies and was harmonised as ‘standard care’ for clarity.

Reporting completeness ranged from 52 % to 93 % of applicable CHEERS items^(5)^ across the twenty-four studies (Table 2). Most studies reported title, comparators and outcomes; however, HEAP, heterogeneity and uncertainty were the least reported items (Table 2). Interventions from twenty-four included studies comprised nutrition education^(21,24,26,27)^, diet (healthy eating) and physical activity (PA) with or without behavioural strategies^(7–9,15,16)^, counselling^(17,18,22)^, home visits or home visits with group sessions^(23,25,28)^, home visits with counselling^(10)^, motivational interviews to promote healthy food and PA habits^(29)^, a web-based tool for menu planning^(11)^, supplementary support to promote breast-feeding^(20)^, telephone support^(13)^ and information booklets and incentives (vouchers) for breast-feeding^(12)^ (Table 1). In addition, scenario analysis approach was used in a prenatal Women, Infants and Children (WIC) coverage^(19)^, breast-feeding initiation ranges^(14)^ and a national breast-feeding strategy^(30)^. Of twenty-four studies, eighteen studies used data from clustered RCT, four from observational studies^(14,16,19,30)^, one from a systematic review^(8)^ and one stated no source of effectiveness evidence^(23)^. Across studies, comparators were described as usual care^(11,14,17,18)^, standard care^(8,16,21,25)^ or routine care^(15)^, representing the continuation of existing practice (Table 1). Comparator terminology varied across studies and was harmonised as ‘standard care’ for clarity (Table 1).

**Table 2:** Summary of reporting quality based on the CHEERS 2022 checklist (reported/applicable items) for the included studies (*n* 24) Table 2 long description.

Study	Country	Type of economic evaluation	Reported items	Not reported items	Not applicable items	Applicable items	Reported/applicable items	%	Key unreported items
Ahmed (2023)	Uganda	CEA	21	6	1	27	21/27	78 %	Health economic analysis plan (HEAP); discount rate
Anokye (2020)	United Kingdom (UK)	CUA	24	2	2	26	26/28	93 %	Approach to engagement with patients and others affected by the study
Bailey (2022)	Australia	CEA	23	4	1	27	23/27	85 %	HEAP; subgroup/scenario analyses
Broekhuizen (2018)	Nine European countries	CUA	22	4	2	26	22/26	85 %	HEAP; effects of engagement with patients and others affected by the study
Chola (2015)	Uganda	CEA, CUA	26	2	0	28	26/28	93 %	HEAP; approach to engagement with patients and others affected by the study
Dodd (2015)	Australia	CEA	21	4	3	25	21/25	84 %	HEAP; effects of engagement with patients and others affected by the study
Doring (2018)	Sweden	CEA	21	4	3	25	21/25	84 %	HEAP; discount rate; valuation of outcomes
Gowani (2014)	Pakistan	CEA	16	11	1	27	16/27	59 %	HEAP; time horizon, discount rate; valuation of outcomes; measurement and valuation of resource and costs
Hays (2014)	Australia	CEA	21	4	1	25	23/25	92 %	HEAP; characterising heterogeneity vary for subgroups
Heckert (2020)	Burundi, Guatemala	CEA	17	4	6	21	17/21	81 %	Title; HEAP; valuation of outcomes; effects of uncertainty
Hoddinott (2012)	UK	CEA	18	8	2	26	18/26	69 %	HEAP; outcomes valuation
Jacklin (2007)	UK	CUA	22	5	1	27	22/27	81 %	HEAP; discounting on costs
Kolu (2013)	Finland	CUA	23	3	2	26	23/26	88 %	HEAP; effects of engagement with patients and others affected by the study
Kolu (2016)	Finland	CEA, CUA	22	5	1	27	22/27	81 %	HEAP
Nianogo (2019)	USA	CBA, CUA	24	4	0	28	24/28	86 %	HEAP; discounting on intervention costs
Perry (2023)	Guatemala	CUA	11	16	1	27	11/27	52 %	HEAP; comparator(s), perspective, time horizon, effect of uncertainty
Poston (2017)	UK	CUA	23	2	3	25	23/25	92 %	HEAP; approach to engagement with patients and others affected by the study
Pugh (2002)	USA	CEA	16	9	2	25	16/25	64 %	HEAP; effects of uncertainty, conflict of interests
Reeves (2021)	Australia	CEA	21	5	2	26	21/26	81 %	HEAP; approach to engagement with patients and others affected by the study; study findings, limitations, generalisability and current knowledge
Rogozinska (2017)	UK	CEA	24	3	1	27	24/27	89 %	HEAP; approach to engagement with patients and others affected by the study
Walters (2016)	Vietnam	CBA	16	10	2	26	16/26	62 %	HEAP; measurement and valuation of outcomes; measurement and valuation of resources and costs
Waters (2006)	Peru	CEA	18	7	3	25	16/20	80 %	HEAP; effects of uncertainty, conflict of interests
Wynn (2017)	South Africa	CEA	18	8	2	26	18/26	69 %	Title; HEAP; effects of uncertainty; conflict of interest
Garcia (2021)	Kenya	CEA, CBA	22	5	1	27	22/27	81 %	HEAP; valuation of outcomes

CHEERS, Consolidated Health Economic Evaluation Reporting Standards; CEA, cost-effectiveness analysis; CUA, cost–utility analysis; CBA, cost–benefit analysis.

Reporting quality was assessed using the CHEERS 2022 checklist. Each item was scored as reported or not reported. Items considered not applicable (e.g. model-based components in trial-based studies) were excluded from scoring. The overall percentage score for each study was calculated as the number of reported items divided by the number of applicable items × 100. Scores are presented as the number of reported items out of applicable CHEERS 2022 checklist items.

The majority of studies (*n* 15) adopted a time horizon of 1 year or shorter, while eight studies used longer time horizons^(10,14,17,19,21,25,26,29)^, and one study did not report the time horizon^(23)^. Additional costs were compared to incremental effects, but they were not always associated with incremental benefits^(21)^. Costs and relevant consequences of the intervention were not always discounted to bring future costs and consequences to the present values^(10,17,21,29)^ (Table 1). Uncertainties were not fully addressed in the form of sensitivity analyses in most studies (Table 2). Due to substantial heterogeneity, quantitative synthesis was not appropriate; therefore, the review focused on methodological approaches. Like interventions have been grouped together to provide an overview of the different forms of intervention for which economic evaluations have been conducted.

### Interventions during pregnancy to improve maternal health

Three studies focused on interventions to improve maternal health during pregnancy including interventions of PA, healthy eating and PA promotion^(7)^, intensified counselling on PA, diet and appropriate weight gain with the subsequent 7-year follow-up compared to usual care^(17,18)^ (Table 1).

A decision-tree approach was applied with a time horizon of less than 1 year (during pregnancy) from the societal perspective with no discounting^(7,18)^, and the societal perspective was supplemented by a healthcare provider perspective in a sensitivity analysis^(7)^. Costs and consequences were not discounted owing to the short time horizon^(18)^. Discounting was not considered, even though the QALY gained was based on a lifetime horizon^(17)^. Other than including sickness absence^(17)^, outcomes were predominantly health outcomes^(7,17)^ that were measured by QALY gained or incremental cost-effectiveness ratio (ICER) for antenatal service, gestational weight gain, fasting blood glucose, insulin resistance and newborn birth weight. HRQoL data were collected using a generic tool, EQ-5D from a multi-centre RCT, valued using a European tariff. Only maternal health outcomes were reported and not longer-term outcomes for the newborns^(7)^. The valuation of consequences using a visual analogue scale and a generic tool 15D to assess HRQoL and produce ICER was difficult to interpret^(17,18)^.

Costs were mainly Dutch healthcare costs that might be inappropriate, as country-specific cost data from a multi-centre study involving nine European countries would provide a better estimate^(7)^. Estimated cost savings in a 7-year follow-up did not consider relevant intervention costs or cost savings from newborn’s health^(17)^, while cost savings in later life from obesity prevention and disturbances in glucose metabolism in childhood were not reported^(18)^.

Linear multi-level analysis using multiple imputation for missing data was undertaken with an unjustified sensitivity analysis^(7)^. Sensitivity analyses were undertaken with doubled intervention costs without justifications^(18)^ and the complete case and standardised productivity costs^(17)^.

### Interventions during pregnancy to improve fetal outcomes

Two studies examined interventions during pregnancy to improve fetal outcomes. Dietary advice, PA and behavioural strategies were compared to standard care^(9)^ (Table 1). The effects of varying coverage rates of the special supplemental nutrition programme for women, infants and children were assessed compared to current practice with an assumed 84 % coverage^(19)^. A ‘do-nothing’ strategy was considered as a comparator by not including explicitly any dietary or lifestyle advice^(9)^ or by including 0 % coverage^(19)^. A decision tree was used with a short time horizon (pregnancy to 6-week postpartum) from a healthcare perspective^(9)^, while a newborn’s life course from pregnancy was modelled from a societal perspective with the discounting rate of 3 % for costs and QALY^(19)^.

Outcomes included cost per additional infant with birth weight above 4 kg prevented, cost per additional infant with respiratory distress syndrome prevented and cost per preterm birth avoided. Health state utility values from pregnancy to 4 months postpartum (follow-up) were derived from a generic instrument, the SF-6D using the thirty-six-item Short Form Health Survey data. The authors described methods to measure differences in health state utility values in the follow-up period between the intervention and control groups; however, the results were not reported^(9)^. Data sources for the willingness-to-pay for a QALY and costs of preterm birth were reported^(19)^.

Potential maternal benefits from the intervention were not modelled given the short time horizon, but future cost savings were supported by reference to literature^(9)^. Costs directly associated with the additional resource use from the time of randomisation until 6 weeks postpartum were included; however, the societal costs reported were not disaggregated, making it difficult to consider different viewpoints^(9)^.

Bootstrapping of patient-level data was used to estimate confidence intervals and to derive cost-effectiveness acceptability curves^(9)^. A range of absolute risk reductions was explored through sensitivity analyses of results, but a potential impact in particular socio-economic groups was not explored^(19)^.

### Interventions to promote breast-feeding

Six studies examined interventions aimed at promoting breast-feeding compared to usual care using a decision tree^(12–14,20,30)^ or a state transition model with three health states (well, diarrhoea and dead (Markov model)^(22)^ (Table 1). The effectiveness of the intervention was established through RCT^(12,13,20,22)^, assumed^(30)^ or derived from different observational datasets^(14)^. Economic questions, PICO and analytical perspectives were stated in a HEAP^(12)^.

The valuation of consequences using cost per DALY averted^(22)^ and the cost per QALY at £20 000 were appropriate for the decision problems posed^(14)^. A provider’s perspective was adopted with some costs borne by the household^(14,30)^, and a societal perspective was adopted where some costs for mothers’ time spent feeding the infant and health service costs were considered^(20)^. DALY were not age-weighted but discounted at an unjustified rate of 3 %, and the discount rates for costs and consequences were varied at 0 % and 6 % in the sensitivity analysis^(22)^. QALY gained from reduced illnesses were discounted at 3.5 %, but it was unclear if the cost savings reported from the literature were discounted^(14)^. Improved infant survival, reduced breast milk substitutes purchases, health service utilisation and health outcomes were identified as important outcomes but were not measured, underestimating full consequences such as improved workforce (productivity) or reduced health service utilisation^(12,13,20,22)^.

Regression models were used to adjust for baseline variables and potential imbalances in the intervention group, with appropriate statistical analysis of patient-level data for estimating the transition probabilities^(12,22)^. Limited statistical analysis was appropriate given the small sample size^(20)^. Although ranges were justified for the one-way sensitivity analysis, no details were provided on the distributions of values for the probabilistic sensitivity analysis^(22)^. Only a threshold analysis was reported with unjustified ranges used^(14)^, while an assumed association between breast-feeding and increased IQ was combined with a relationship between IQ and earnings without testing such assumptions^(30)^. The interpretation of cost-effectiveness appeared appropriate; however, it was unclear whether the reported results were valid due to the assumed intervention effectiveness^(30)^. The incremental cost per change in breast-feeding is difficult to interpret when the value of increased breast-feeding is not explicitly stated^(13)^. The probability of the intervention being cost-effective at different willingness-to-pay levels for an additional breastfed baby was estimated among socio-economically disadvantaged groups in the National Health Service (NHS), but it was unclear if the findings could be applied to a broader population^(12)^.

### Interventions to improve early childhood development

Three studies used simple decision trees to evaluate the cost-effectiveness of interventions designed to improve ECD compared to the status quo^(28)^ or current practice^(21,26)^. Costs and consequences were discounted at 5 % for the CBA^(28)^, while no discounting was undertaken for the interventions over 18–24 months^(21,26)^ (Table 1). The pathway for delivering the intervention was outlined, ensuring the health outcomes were assessed^(21)^.

The progress and changes of ECD were measured using the Bayley Scales of Infant and Toddler Development – III (BSID-III)^(21,26,28)^; however, these measures did not capture other relevant costs and consequences beyond ECD. The effectiveness of intervention was established through RCT^(21,26)^. An 18-month time horizon with a focus on cognitive scores might not be wide enough for the decision problem from the perspectives of providers and mothers^(21)^. The only effect considered was ECD using the BSID-III, while all costs to the service providers were considered^(21,26)^, although incorporating costs incurred by mothers in addition to the public perspective would have been better for decision-making.

Costs to service providers^(21,26)^ and mothers’ time and costs of additional schooling were considered^(28)^. Future costs beyond 18 months and consequences^(21)^ and the costs to mothers^(26)^ were identified but not reported. No attempts were made to explore the value of child development as measured using BSID-III^(21,26,28)^. Costs may be incremental, but the effectiveness measure using BSID-III used in the reported cost-effectiveness ratio was not incremental^(26)^. Threshold analyses and deterministic sensitivity analysis were undertaken to address uncertainty in the estimates of costs and consequences^(28)^. A regression analysis and a probabilistic sensitivity analysis^(21)^ or limited sensitivity analysis were conducted to address uncertainties^(26)^. An additional sd per 100 US dollars is not readily interpretable for decision-making^(28)^. The future benefits of ECD from the maternal nutrition intervention, such as academic attainments, resources for the provision of school dropouts and productivity (earnings) gains/losses were not explored^(21,26,28)^.

### Interventions to improve maternal and child health

Five studies examined interventions to improve maternal and child health among pregnant women compared to standard or routine care from the provider or the NHS perspective^(8,15,16,25)^ or without stating a comparator or a perspective^(23)^. A decision tree was used to estimate costs and consequences at a cohort level^(8,15,16,25)^ (Table 1).

Effectiveness was established through RCT^(15,25)^ or a meta-analysis of individual-level data^(16)^. The impact on child and maternal mortality was estimated indirectly using a relationship mortality and increased coverage of the interventions^(23)^. The effectiveness of intervention was based on literature where baseline data were from a representative dataset^(8)^. The reference to the literature to justify the value of the outcomes achieved appeared unsystematic, and the within-trial analysis provided only costs and consequences up to the delivery of baby^(25)^.

Costs per complication avoided were estimated by utilising the meta-analysis of patient data with six identified clinical pathways^(16)^. Admissions to neonatal intensive care unit were included without other health outcomes for the child^(8)^, or only costs and outcomes during pregnancy were considered^(15)^. The valuation of consequences was appropriate where the analysis focused on the cost per additional case prevented or the QALY estimated from EQ-5D^(8,15)^.

Resource utilisation was described and justified from the RCT or literature^(8,15,16,25)^; however, the broader maternal and neonatal benefits and related costs were omitted beyond pregnancy^(8,15,16)^. Any lives saved in under-fives or mothers were assumed to live without any disability, but this assumption was not explored in a sensitivity analysis^(23)^. The source of costs and consequences were clearly stated^(15,25)^. An appropriate statistical analysis was applied to patient-level data^(15,16)^, and repeated analyses were performed with low and high estimates of the outcomes^(25)^. However, only one study specified distributions for all the variables in the probabilistic sensitivity analysis^(16)^. The most deprived group was compared to less deprived groups in a subgroup analysis^(15)^.

### Interventions to reduce child stunting

Two studies assessed the costs and consequences of interventions aimed at reducing stunting compared to usual practice using a decision tree from the provider perspective^(24)^ and from the provider perspective plus a degree of societal perspective^(27)^ (Table 1). A 3 % discount rate was applied to costs and consequences over 2 years^(24)^, while no discounting was applied owing to the 1-year time horizon^(27)^. Although a wide range of impacts was collected in a multi-arm cluster RCT, only detailed programme costs were reported^(24)^. The utilisation of health services, well-baby check-ups, nutritional status and percentage of stunting were reported from a cluster RCT^(27)^.

An activity-based costing approach was used, but the multisectoral nature of the programme^(24)^ or the chosen 1-year time horizon^(27)^ made relevant measurement of stunting prevention challenging. The costs of intervention for stunting prevention occur within the chosen 1- or 2-year time horizon^(24,27)^. The cost per percentage point reduction in stunting was not readily interpretable which was not incremental costs compared to incremental effects^(24)^. No sensitivity analysis was undertaken^(24,27)^. None of the studies provided the generalisability of reported results to other settings. Reduced stunting may have wider benefits, but such benefits were not considered. The valuation for the reported cost per case of stunting prevented was appropriate for the decision problem, in which incremental costs were compared to incremental effects; however, the willingness-to-pay remained unknown^(27)^.

### Interventions to prevent early childhood obesity

Two studies estimated the cost-effectiveness of motivational interviews, home visits and counselling to prevent early childhood obesity among first-time parents and children using a decision tree, compared to usual or standard care^(10,29)^ (Table 1). Effectiveness data came from cluster RCT, and BMI at age 4 years and a unit BMI avoided were primary outcomes^(10,29)^. No other information was collected or modelled on health consequences for the children, especially from the chosen societal perspective^(29)^. Costs for intervention and healthcare including downstream costs due to healthcare utilisation of the children were considered from the health funder’s perspective^(10)^. There were no attempts to value a unit decrease in BMI or BMI-*z* or any consequences of such a change^(10,29)^. QALY estimation for children can be challenging, and the unknown willingness-to-pay for preventing BMI increases in childhood made the reported cost per BMI unit prevented difficult to interpret^(29)^. Only costs were discounted at a justified rate of 5 %^(10)^.

Appropriate non-parametric bootstrapping was used to address uncertainties in costs and consequences and the cost-effectiveness acceptability curves^(10,29)^. Parameter uncertainties were addressed by halving of meeting times^(29)^ or using lower costs^(10)^. Motivational interviews offered to promote healthy eating and PA habits among families with infants were embedded in the existing child healthcare centres that might facilitate the adoption and sustainability of newly introduced intervention^(29)^. The results were reported to be generalisable as most population attend the regular child healthcare centre meetings in Sweden^(29)^, while the RCT recruited from antenatal clinics in socio-economically disadvantaged areas of Sydney, which limits the generalisability of results^(10)^.

### Intervention to improve the implementation of dietary guidelines

One study assessed the cost-effectiveness of a web-based tool for menu planning to improve the implementation of dietary guidelines compared to usual care over one year^(11)^ (Table 1). Effectiveness data were taken from a cluster RCT that focused on the compliance of dietary guidelines. Resource utilisation and consequences were taken from a trial, but relevant and identified consequences including dietary behaviour of children were omitted from the measurement. The cost of feeding children or relevant consequences were omitted. The non-significant increase in the mean number of food groups that were compliant with the guidelines was based on a 1-week data collection, and the unclear value of increased compliance made it difficult to measure the intervention effectiveness.

## Discussion

The aims of this review are to identify economic evidence and evidence gaps relevant to early-life interventions to promote healthy eating within the first 1000 days, and the findings will inform the future economic evaluation of relevant interventions. This review highlights gaps in the evidence and opportunities for strengthening future economic evidence for early-life interventions to promote healthy eating to better inform decision-making. Overall, the twenty-four studies demonstrated mixed quality (Tables 1 and 2). Notably, four studies presented relatively transparent methods with the interpretation of CUA results being helpful for decision-making^(12,14–16)^, while remaining studies claimed the cost-effectiveness or cost savings of interventions without clearly specifying the decision context. Most studies identified the PICO excluding one study^(23)^. Reduced breast milk substitutes purchase, health service utilisation, earnings, strengthened workforce, reduction in NCD and changes in HRQoL were identified as important outcomes but were not measured in the studies mainly due to the short time horizon chosen^(7–10,12,13,15,16,18–20,22,27,30)^. Additionally, the challenges in QALY estimation among children were recognised^(29)^. The CHEERS checklist^(5)^ was applied to assess reporting quality; however, this does not necessarily reflect the methodological quality or decision relevance of the included studies. Therefore, reported CHEERS scores (Table 2) should be interpreted with caution, as they represent ‘adherence to reporting standards rather than the overall quality of reporting’^(5)^.

Only one study had a HEAP, examining the within-trial cost-effectiveness of incentivised breast-feeding alongside a cluster RCT^(12)^. A HEAP should be an integral part of a study protocol, outlining economic questions, PICO and analytical perspectives^(12)^. Subsequently, the type of economic evaluation with the most appropriate outcome measures and valuation can be determined, leading to the identification and collection of relevant data for estimating the cost and consequences compared to relevant comparators. Uncertainties arise from the chosen modelling approach and parameters, and appropriate approaches to handling uncertainties should be part of a HEAP.

A decision tree was mostly connected with a short time horizon, which captured immediate costs and consequences of interventions. Of the twenty-four studies, early-life nutrition interventions were mostly embedded in complex interventions except for three studies^(9,11,12)^. It is encouraging to see mother–child dyads as the study populations^(10,12,14,21,22,24,28)^, as this approach facilitates the capture of the costs and benefits of interventions for both mothers and children. The expected place of interventions in the care pathway was clearly stated, which helped understanding of the interactions in different stages of development. Healthy eating in relation to weight management during pregnancy and relevant complications such as gestational diabetes mellitus or maternal behaviour changes would have consequences beyond pregnancy, but these were not considered^(10,21,28)^. Several studies^(7–9,12,15,17,18,20–22,24,26,29)^ were limited to narrowly identified outcomes with a short time horizon (6–12 months) that subsequently failed to capture the full benefit of interventions, for example, prevention of maternal and childhood obesity, and reduced NCDs. Future economic evaluation may be able to include relevant outcomes specific to the different interventions or different sequences and which may subsequently improve the cost-effectiveness of interventions to promote healthy eating behaviours. Early-life interventions to promote healthy eating habits in the first 1000 days may be expected to have particularly strong effects among socially vulnerable population groups. However, the included studies did not provide sufficient data to allow exploration of heterogeneity by socio-economic status. This highlights an important gap in the existing evidence base. Future research should prioritise the inclusion and reporting of socio-economic characteristics to enable assessment of differential effects in different population groups and inform more equitable decision-making.

This review has several limitations. Reported results were not aggregated, reported costs were not presented to a common monetary value/year or a meta-analysis of reported results was not undertaken due to the heterogeneity of reported outcomes and used methods. A key word, e-learning (digital tools), was not included in the search as a focused search would retrieve studies with a narrow range resulting in missing relevant studies. This was related to the findings from the review, which indicated that most early-life nutrition interventions to promote healthy eating were part of complex interventions. Like any validated checklists, the chosen checklists for this review have limitations^(5,6)^, with less focus on capturing intersectoral costs and consequences^(31)^. The (un)intended consequences of public health interventions are not always evenly distributed across the population, and understanding the impact of interventions by segmenting the population would offer better insights to decision-making. The search was limited to the English language, potentially excluding relevant studies published in other languages. Reference cases outline a set of principles that set out the range of methodologies deemed most appropriate for a decision context when undertaking an economic evaluation of health interventions^(32)^. However, when those principles are applied to the economic evaluation of public health interventions, including nutrition interventions that are often complex, a longer time horizon is required to capture relevant costs and (un)intended consequences^(33,34)^.

Overall, this review provides evidence to support improvements in the future economic evaluation of interventions to promote healthy eating in the first 1000 days. Most of all, a HEAP should be developed and specified in advance. Healthy eating habits established during the first 1000 days can influence dietary habits later in life with potentially important implications for health conditions such as diabetes and obesity. Long-term or recurrent health events, such as diabetes, would be better represented using a pathway modelling with defined health states, moving through different stages over time (Markov states)^(33)^. Key elements of early-life interventions to promote healthy eating can be mapped along the ECD stages to inform economic evaluation with a longer time horizon, enabling the capture of costs and outcomes. Pathway modelling links short- and medium-term outcomes, for example, connecting early-life nutrition interventions and breast-feeding promotion^(12,13,20,22,25)^ to ECD outcomes^(21,26)^, as well as later behaviours such as healthy eating, later diet and PA at the age of 4 years^(29)^. These pathways may further develop into longer-term health outcomes, including diabetes, obesity and related NCDs, enabling a more comprehensive assessment of costs and full benefits. Furthermore, a 7-year follow-up study considering longer time horizon in the analysis^(17)^ and a study including a HEAP^(12)^ demonstrated the possibility of designing a study from the outset incorporating a long-term follow-up with a HEAP. The analytical perspectives and the decision context with respect to resource allocation should be stated^(12,14–16)^.

The comparator ‘standard care’ is not necessarily the same across different studies/settings.

‘Usual care’ was described as reflecting what would happen in routine practice^(10)^. Nutrition education interventions are commonly embedded as part of complex interventions, adding further challenge to defining the comparator. Studies adopted ‘status quo’, ‘standard care’, ‘standard practice’ or ‘usual care’ as a comparator but rarely provided detail. However, ‘standard care’ as a comparator cannot be assumed the same across different studies/settings. Furthermore, the estimated cost-effectiveness of a nutrition intervention compared to ‘standard care’ might not be helpful in the decision-making context, especially a multi-centre trial in nine European countries because the definition of ‘standard care’ is unlikely to be the same in each country^(7)^. ‘Standard care’ should be clearly described along with the intervention in economic evaluation. It is not known whether the chosen ‘standard care’ comparators were cost-effective alternatives in the included studies. Having a HEAP offers an opportunity to clearly identify the relevant comparators^(5,6)^. In two studies, relevant comparators were identified through a systematic evidence review^(15,16)^, representing a more robust approach.

Short- and medium-term outcomes should include the changes in the quantity of fruits and vegetables eaten weekly (or monthly), weight and height measurements, maternal weight changes, gestational diabetes mellitus status, ECD^(21,26)^ and a unit change in BMI linked to healthy eating^(17,29)^. Long-term outcomes should extend beyond immediate health effects to include maternal and child obesity, measured through longitudinal BMI tracking and the incidence of obesity and related NCD. Future research should also prioritise broader outcomes, including school attainment, cognitive development, employment and income trajectories, and psychosocial and mental health outcomes. Measuring HRQoL using validated questionnaires such as EQ-5D and deriving health state utility values for a pathway modelling with Markov states would be recommended^(7,9,12,15,19)^. Nutrition interventions to promote healthy eating tended to be embedded in a complex intervention such as intensive counselling by specially trained professionals that may be more effective but also cost more, adding further complexity to clearly defining relevant comparators^(10,17,35)^. Costs from the health service utilisation, costs of providing healthy eating environments, academic attainments, resources for the provision for school dropouts and productivity (earnings) gains/losses could be captured to demonstrate the costs and consequences. Uncertainties arising from the data sources should be addressed through sensitivity analyses for the robustness of reported results.

Several studies (*n* 13) had narrowly defined outcomes and short time horizon with unclear decision context for the evaluation of early-life nutrition interventions to promote healthy eating in the first 1000 days, making the reported results less helpful in decision-making. Four CUA stated the perspective of analysis with clear decision rules, making the results useful in decision-making^(12,14–16)^. The findings from this review may offer important insights to inform the design and planning of future economic evaluation of early-life nutrition interventions to promote healthy eating. Future economic evaluation should include relevant comparators both separately and in combination, such as intensified counselling, home visits or different combinations of comparators to better inform decision-making and resource allocation.

### Conclusion

This review identified consistent methodological challenges in the economic evaluation of early-life nutrition interventions. Comparators were inconsistently defined, with the varied definition of ‘standard care’ across studies and settings, limiting comparability and decision relevance. Furthermore, most studies adopted short time horizons, often one year (or shorter), limiting the ability to capture longer-term outcomes such as obesity, educational attainment and life-course health impacts. Chosen outcomes were narrow, focusing on immediate health outcomes without consideration of broader or downstream effects. In addition, socio-economic characteristics or exploring differential effects across population groups were rarely reported in the included studies. Addressing these identified issues requires more robust and transparent approaches to economic evaluation. Incorporating a HEAP as an integral part of a study protocol ensures that key methodological elements (such as the economic question, PICO, analytical perspectives and the approaches to handling uncertainties) are pre-specified and aligned with decision contexts to support evidence-based decision-making.

## Supporting information

10.1017/S1368980026103061.sm001 Jeong and Cairns supplementary material Jeong and Cairns supplementary material

## Supplementary material

For supplementary material accompanying this paper visit https://doi.org/10.1017/S1368980026103061

## Table 1 Long description

The table presents a summary of included studies on economic evaluations of interventions for pregnant women. It has 24 rows and 7 columns. The columns are labeled as Study, Country, Population, Intervention, Comparator, Outcome, and Type of economic evaluation. Each row provides details for a specific study, including the study ID, country where the study was conducted, the population studied, the intervention applied, the comparator used, the outcome measured, and the type of economic evaluation performed. The studies cover various countries and interventions, with the most common type of economic evaluation being cost-effectiveness analysis (CEA).

Navigate back to Table 1.

## Table 2 Long description

A table summarizing the reporting quality of 24 studies based on the CHEERS 2022 checklist. The table has 24 rows and 8 columns. The columns are labeled Study, Country, Type of economic evaluation, Reported items, Not reported items, Not applicable items, Applicable items, Reported/applicable items %, and Key unreported items. Each row lists the details for a specific study, including the study name, country, type of economic evaluation, number of reported items, not reported items, not applicable items, applicable items, percentage of reported/applicable items, and key unreported items. The studies are from various countries and cover different types of economic evaluations such as CEA, CUA, and CBA. The percentage of reported/applicable items ranges from 52% to 93%. Key unreported items include health economic analysis plan, discount rate, approach to engagement with patients and others affected by the study, and more.

Navigate back to Table 2.
