# Supplementary material for: Systematic review of economic evaluation of early-life nutrition interventions to promote healthy eating during the first 1000 days
Source: Public Health Nutr. 2026 Jul 13;29(1):e173. doi: 10.1017/S1368980026103061 (PMC13740159; doi:10.1017/S1368980026103061)
Supplement: Supplementary file 1 [file S1368980026103061sup001.docx]

**Appendix 1. Review protocol**

| Review question | Systematic review of economic evidence of early-life nutrition interventions to promote healthy eating in the first 1000 days |
| --- | --- |
| Type of review question(s) | Cost-effectiveness |
| Objective of the review | To identify economic evidence and methodological approaches used in evaluation of early-life interventions to promote healthy eating in the first 1000 days in community, education, and home settings and establish evidence gaps. |
| Eligibility criteria - population/disease/condition/issue/domain | Pregnant women, infants and young children 0-2 years olds at home, education, and community settings |
| Eligibility criteria - intervention(s)/exposure(s)/prognostic factor(s) | Activities such as education, feeding or dietary practice to promote healthy eating. Healthy eating includes breastfeeding, daily consumption of grains, protein, dairy, fruits and vegetables while limiting intake of processed foods, saturated and trans fats, limiting daily salt and sugar intake. |
| Eligibility criteria - comparator(s)/control or reference (gold standard) | Usual care and other existing interventions |
| Outcomes and prioritisation | Primary outcomes - improved frequency of healthy diet (for example, changes in weekly consumption of fruits and/or vegetables, starting and continuing breastfeeding, BMI, BMI-z score, height, weight, existing anthropometric measures  Secondary outcomes - health-related quality of life, disability-adjusted life year |
| Eligibility criteria - study design | - Cost-utility (cost per quality-adjusted life years gained or incremental cost-effectiveness ratios) - Cost benefit (benefit: cost ratio) - Cost-effectiveness (cost per unit of effect) - Cost-consequences |
| Other inclusion exclusion criteria | Inclusion criteria   - Economic evaluation of early-life nutrition interventions to promote healthy eating (breastfeeding, food or diet, eating, nutrition, fruit or vegetable, feeding or dietary practices/programmes) for the first 1000 days - Peer-reviewed and published in English language - Full-text publications from year 2000 - Home or education/community settings   Exclusion criteria   - Title/abstract that are irrelevant to nutrition interventions for the first 1000 days - Conference abstracts or study protocols with no full publication of economic evaluation - Unspecified or not clearly defined early-life nutrition interventions - Nutrition interventions for clinical diagnosis - Interventions for children older than 2 years or non-pregnant women - Interventions related to clinical diagnosis, food (in)security, malnutrition, food fortification, micronutrients, home parenteral/enteral feeding |
| Proposed sensitivity/ sub-group analysis, or scenario analysis | Not applicable |
| Selection process - duplicate screening/ selection/ analysis | The review will use the priority screening function, EndNote.  Double screening will be carried out for 10% of titles and abstracts by the second reviewer. Disagreements will be resolved by discussion. Inter-rater reliability will be assessed and reported where applicable.  The study inclusion and exclusion lists will be checked with members of the Nutrition Now to ensure no studies are excluded inappropriately. |
| Data management (software) | EndNote® will be used:   - To store lists of citations - To sift studies based on title and abstract - To record decisions about full text papers - To order freely available papers via retrieval function - To request papers via UiA information services - To store extracted data   STATA® and/or excel® will be used to perform further analyses, where appropriate. |
| Information sources - databases and dates | The purpose of the search is to identify the best available evidence to address the questions without producing an unmanageable volume of results.  The following methods will be used to identify the evidence:   - The databases listed below will be searched with an appropriate strategy. - The websites listed below will be searched or browsed with an appropriate strategy.   **Databases strategies**  The database strategy will be adapted as appropriate from the most recent relevant publication, where possible, taking into account the resources available to this review, the subscriptions that UiA has, in line with the indexing policies and the final scope for the economic evaluation of Nutrition Now.  The search strategy will take this broad approach: nutrition interventions, community setting, 1000 days, children AND economic evaluation will be searched separately according to the individual Review Protocols. Full-text studies in English languages published from 2000 to current studies will be included.  Feedback on the principal database strategy was sought from key members of Nutrition Now.  The principal search strategy will be developed in MEDLINE (Ovid interface) and then adapted, as appropriate, for use in the other sources listed, taking into account their size, search functionality and subject coverage.  The other databases will be:   - Web of Science   **Cost effectiveness evidence**  The following databases will be searched with agreed study-type search filters applied to a strategy based on the one in a separate search strategy:  • Embase via Ovid  • MEDLINE via Ovid  In addition, the following sources will be searched without study filters:  • EconLit via Ovid  • HTA database via CRD <https://www.crd.york.ac.uk/CRDWeb/> via CRD <https://www.crd.york.ac.uk/CRDWeb>  **Website searching**  The following websites will be searched with an appropriate strategy and the first 50 results examined to identify any UK reports or publications relevant to the review that have not already been identified: The following websites will be searched with an appropriate strategy and the first 50 results examined to identify any UK reports or publications relevant to the review that have not already been identified:  • WHO Increasing fruit and vegetable consumption to reduce the risk of noncommunicable diseases <https://www.who.int/tools/elena/interventions/fruit-vegetables-ncds>)  • Google (restricting to the UK domains) [www.google.co.uk](http://www.google.co.uk)  • Google Scholar [www.scholar.google.com](http://www.scholar.google.com)  • NICE Evidence Search <https://www.evidence.nhs.uk>  Searches will also be conducted on the following key websites for relevant Norway reports or publications:   - Norwegian Institute of Public Health (NIPH) - Department of Health Norway   **Database search limits**  Database functionality will be used, where available, to exclude:   - non-English language papers - animal studies - editorials, letters and commentaries - conference abstracts and posters - registry entries for ongoing or unpublished clinical trials - duplicates - sources will be searched from 2000 to current.   **Quality assurance**  The guidance from the Information Service team at UiA will quality assure the principal search strategy and peer review the strategies for the other databases.  Any revisions or additional steps will be agreed by the economic evidence review team before being implemented. Any deviations and a rationale for them will be recorded alongside the search strategies.  **Search results**  The database search results will be downloaded to EndNote before duplicates are removed using automated and manual processes. The de-duplicated file will be exported in RIS format for data screening using an appropriate software i.e. EPPI-Reviewer, Microsoft Excel®. |
| Highlight if amendment to previous protocol | Not applicable |
| Search strategy - for one database | See search strategy for Medline |
| Data collection process - forms/duplicate | The literature will be searched to identify economic evidence of early-life nutritional interventions for healthy eating the first 1000 days in the community, education and home settings. Medline, Embase (2000-1 October 2023) and Health Economic Evaluation Database (HEED) will be searched using the keywords early-life nutrition interventions, healthy eating, first 1000 days, economic evaluation. The search will be restricted to studies in English. The search will be broadened to include websites of the National Norwegian Public Health authority, NICE ([www.nice.org.uk](http://www.nice.org.uk)) to minimise the chance of missing relevant studies. Economic filters will be applied for the search of Medline and EMBASE.  A simplified search will be undertaken without economic filters for evidence on economic databases such as Econlit or HEED.  The following websites will be searched with an appropriate strategy and the first 50 results examined to identify any relevant reports or publications relevant to the review that have not already been identified:  • Google (restricting to UK domains) [www.google.co.uk](http://www.google.co.uk)  • Google Scholar [www.scholar.google.com](http://www.scholar.google.com)  Relevant conference abstracts will be tracked for full publications. All search results will be downloaded into EndNote and duplicates will be removed. Titles and abstracts will be screened between two independent reviewers and full papers that did not meet the inclusion criteria will be excluded.  Full text will be acquired for the remaining studies, studies with insufficient details from the abstracts for review-decision making will be also included.  All included studies will be read, and any disagreements will be resolved by discussion between the two reviewers. |
| Data items - define all variables to be collected | Reviewers to use existing table/format i.e. Drummond checklist (2005) |
| Methods for assessing bias at outcome/study level | The consolidated health economic evaluation reporting standards (CHEERS 2022) checklist was used to assess reporting quality  [Husereau D, Drummond M, Augustovski F, de Bekker-Grob E, Briggs AH, Carswell C, et al. Consolidated Health Economic Evaluation Reporting Standards 2022 (CHEERS 2022) Statement: Updated Reporting Guidance for Health Economic Evaluations. Appl Health Econ Health Policy. 2022;20(2):213-21]  A quality appraisal criterion (Drummond 2005) will be used for quality appraisal of included economic evidence. [Drummond M, Sculpher M, Torrance G, O'Brien B, Stoddart G. Methods for the economic evaluation of health care programs. Oxford: Oxford University Press; 2005.] |
| Criteria for quantitative synthesis (where suitable) | Incremental cost-effectiveness ratio and/or cost-benefit ratio, where suitable |
| Methods for analysis – combining studies and exploring (in)consistency | Narrative |
| Sources of funding/support | Norwegian Research Council (Reference number: 320521)  UiA |
| PROSPERO registration number | Not Applicable |

**Appendix 2. Search results**

**Appendix 2.1 Ovid Medline** ® ALL <1946 to April 11 2024>, EMBASE <1974 to 2024 Week 14>

|  | **Searches** |  | **Results** |
| --- | --- | --- | --- |
| 1 | (food or diet or eat or eating or nutrition* or fruit* or vegetable* or feeding or dietary or beverage or breastfeeding or "breast feeding" or menu or meal or meals or mealtime or dinner or breakfast or lunch*).ti,ab,hw,kf. |  | 4,771,751 |
| 2 | exp Sugar-Sweetened Beverages/ use medall |  | 1,048 |
| 3 | exp sweetened beverage/ use oemez |  | 4,587 |
| 4 | or/1-3 |  | 4,771,901 |
| 5 | (intervention* or promot* or program* or trial* or randomi* or implement*).ti,ab,kf,hw. or (randomly or experimental* group* or quasi experimental*).ti,ab. or Randomized Controlled Trial.pt. or (control* adj2 (stud* or group*)).ti,ab,kf,hw. |  | 22,924,872 |
| 6 | exp economics/ use medall |  | 673,060 |
| 7 | (health economics/ or exp economic evaluation/) use oemez |  | 393,177 |
| 8 | (cost* adj3 (analys* or benefit* or comparison* or consequence* or effective* or evaluat* or measure* or minium* or util*)).ti,ab,kf. |  | 617,455 |
| 9 | (economic adj2 (analys* or benefit* or comparison* or evaluat* or measure* or model* or review*)).ti,ab,kf. |  | 98,276 |
| 10 | ((financ* or money or monies or monetary or budget*) adj2 expenditure*).ti,ab. |  | 1,615 |
| 11 | (value adj2 (money or monetary)).ti,ab. |  | 7,419 |
| 12 | or/6-11 |  | 1,460,195 |
| 13 | (day care or daycare or childcare or early child education* or nursery or nurseries or kindergarten* or ecec).ti,ab,hw,kf. |  | 92,812 |
| 14 | ((child* or educat*) adj3 (service* or setting* or care)).ti,ab. |  | 225,215 |
| 15 | (parent* or famil* or mother* or father* or maternal* or paternal* or home or homebased or homehealth* or homecare* or communit* or municipal* or primary care or primary health*).ti,ab,hw. |  | 7,947,165 |
| 16 | catering service/ use oemez |  | 21,812 |
| 17 | Food Services/ use medall |  | 6,222 |
| 18 | (food adj1 (service* or catering)).ti,ab. |  | 5,138 |
| 19 | or/13-18 |  | 8,114,535 |
| 20 | (newborn* or infant* or infancy or baby or babies or toddler* or preschooler* or pre-schooler* or pre schooler* or child*).ti,ab,hw. |  | 7,692,758 |
| 21 | (first adj3 (day* or month* or year)).ti,ab. |  | 625,543 |
| 22 | or/20-21 |  | 8,156,551 |
| 23 | 4 and 5 and 12 and 19 and 22 |  | 7,290 |
| 24 | (conference abstract or letter or editorial).pt. |  | 9,152,458 |
| 25 | 23 not 24 |  | 6,575 |
| 26 | limit 25 to english language |  | 6,335 |
| 27 | limit 26 to yr="2000 -Current" |  | 5,163 |
| 28 | remove duplicates from 27 |  | 3,946 |
| 29 | limit 28 to human |  | 3,742 |

**Appendix 2.2 Econlit (EBSCO*host*)**

| **Search elements** | **#** | **Query** | **Limiters/Expanders** | **Results** |
| --- | --- | --- | --- | --- |
| Nutrition… | S1 | food OR diet OR eat OR eating OR nutrition* OR fruit* OR vegetable* OR feeding OR dietary OR beverage OR breastfeeding OR "breast feeding" OR menu OR meal OR meals OR mealtime OR dinner OR breakfast OR lunch* | Search modes - Boolean/Phrase | 69,070 |
| Child, newborn, infant…  N2 = adj3 in Ovid = NEAR/2 in WoS syntax | S2 | newborn* OR infant* OR infancy OR baby OR babies OR toddler* OR preschooler* OR "pre-schooler*" OR "pre schooler*" OR child* OR (first N2 (day* OR month* OR year*)) | Search modes - Boolean/Phrase | 57,256 |
| Community settings | S3 | "day care" OR daycare OR childcare OR "early child education*" OR nursery OR nuseries OR kindergarten* OR ecec OR parent* OR famil* OR mother* OR father* OR maternal* OR paternal* OR home OR homebased OR homehealth* OR homecare* OR communit* OR municipal* OR "primary care" OR "primary health*" OR "food service*" OR "catering service*" OR ((child* or educat*) N2 (service* or setting* or care)) | Search modes - Boolean/Phrase | 161,019 |
| Intervention, implementation studies | S4 | intervention* OR promot* OR program* OR trial* OR randomi* OR implement* OR randomly* OR (control* N1 (stud* or group*)) | Search modes - Boolean/Phrase | 270,452 |
| Combining the search elements | S5 | S1 AND S2 AND S3 | Search modes - Boolean/Phrase | 3,260 |
| ***Limit year*** | ***S7*** | ***S1 AND S2 AND S3*** | ***Limiters - Published Date: 20000101- Search modes - Boolean/Phrase*** | ***3,027*** |

**Appendix 2.3 Web of Science Core collection,** following databases: Science Citation Index Expanded, Social Sciences Citations Index. Use Advanced search screen. April 19 2024

| **Search** | **Results** |
| --- | --- |
| 1: TS=(food OR diet OR eat OR eating OR nutrition* OR fruit* OR vegetable* OR feeding OR dietary OR beverage OR breastfeeding OR "breast feeding" OR menu OR meal OR meals OR mealtime OR dinner OR breakfast OR lunch*) | 3,031,753 |
| 2: TS=(intervention* or promot* or program* or trial* or randomi* or implement* OR (control* NEAR/2 (stud* or group*))) | 8312461 |
| 3: TS=(cost* NEAR/2 (analys* OR benefit* OR comparison* OR consequence* OR effective* OR evaluat* OR measure* OR minium* OR utilit*)) OR TS=(economic NEAR/2 (analys* OR benefit* OR comparison* OR evaluat* OR measure* OR model* OR review*)) OR TS=((financ* OR money OR monies OR monetary OR budget*) NEAR/2 expenditure*) | 531,911 |
| 4: (TS=("day care" OR daycare OR childcare OR "early child education*" OR nursery OR nuseries OR kindergarten* OR ecec OR parent* OR famil* OR mother* OR father* OR maternal* OR paternal* OR home OR homebased OR homehealth* OR homecare* OR communit* OR municipal* OR "primary care" OR "primary health*" OR "food service*" OR "catering service*") OR TS= ((child* OR educat*) NEAR/2 (service* OR setting* OR care))) | 4,657,160 |
| 5: TS=(newborn* OR infant* OR infancy OR baby OR babies OR toddler* OR preschooler* OR "pre-schooler*" OR "pre schooler*" OR child* OR (first NEAR/2 (day* OR month* OR year*))) | 2,798,105 |
| 6: #5 AND #4 AND #3 AND #2 AND #1 | 1,459 |

**Appendix 3. Excluded studies**

| **Study** | **Reasons for exclusion** |
| --- | --- |
| Drago, R., The WIC Program: An Economic Analysis of Breastfeeding and Infant Formula. BREASTFEEDING MEDICINE, 2011. 6(5): p. 281-286. | Exclude - not EE |
| Frick, K.D., et al., Nurse-supported breastfeeding promotion: A framework for economic evaluation. Nursing Economics, 2005. 23(4): p. 165. | Exclude - not EE |
| Gertler, P., Do Conditional Cash Transfers Improve Child Health? Evidence from PROGRESA’s Control Randomized Experiment. Am Econ Rev, 2004. 94(2): p. 336-41. | Exclude - not EE |
| Kumar, D., A.K. Aggarwal, and R. Kumar, The effect of interrupted 5-day training on Integrated Management of Neonatal and Childhood Illness on the knowledge and skills of primary health care workers. Health policy and planning, 2009. 24(2): p. 94-100. | Exclude - irrelevant intervention / outcomes |
| Morrell (2000a) BMJ Costs and effectiveness of community postnatal support workers: randomised controlled trial. Bmj, 2000. 321(7261): p. 593-8. | Exclude - irrelevant intervention |
| Morrell (2000b) HTA Costs and benefits of community postnatal support workers: a randomised controlled trial. Health Technol Assess, 2000. 4(6): p. 1-100. | Exclude - irrelevant intervention |
| Nguyen, T.T., et al., Community support model on breastfeeding and complementary feeding practices in remote areas in Vietnam: implementation, cost, and effectiveness. International Journal for Equity in Health, 2021. 20(1): p. 121. | Exclude - not EE; irrelevant outcomes (reach and coverage) |
| Nkonki, L.L., et al., Costs of promoting exclusive breastfeeding at community level in three sites in South Africa. PLoS ONE, 2014. 9(1): p. e79784. | Exclude - not EE, costing analysis |
| Pil, L., et al., Establishing a method to estimate the cost-effectiveness of a kindergarten-based, family-involved intervention to prevent obesity in early childhood. The ToyBox-study. Obesity reviews : an official journal of the International Association for the Study of Obesity, 2014. 15 Suppl 3: p. 81-9. | Exclude - irrelevant population |
| Pokhrel, S., et al., Potential economic impacts from improving breastfeeding rates in the UK. Archives of Disease in Childhood, 2015. 100(4): p. 334. | Exclude - not EE |
| Shaheen, R., et al., Cost-effectiveness of invitation to food supplementation early in pregnancy combined with multiple micronutrients on infant survival: Analysis of data from MINIMat randomized trial, Bangladesh. BMC Pregnancy and Childbirth, 2015. 15(1): p. 125. | Exclude - irrelevant intervention, not healthy eating |
| Tiruneh, G.T., C.B. Shiferaw, and A. Worku, Effectiveness and cost-effectiveness of home-based postpartum care on neonatal mortality and exclusive breastfeeding practice in low-and-middle-income countries: A systematic review and meta-analysis. BMC Pregnancy and Childbirth, 2019. 19(1): p. 507. | Exclude - not EE |
| Washio, Y., et al., Incentive-based intervention to maintain breastfeeding among low-income Puerto Rican mothers. Pediatrics, 2017. 139(3): p. e20163119. | Exclude - not EE |

**Appendix 4. Glossary**

| **Term** | **Definition** |
| --- | --- |
| 15D | 15-dimensional health related quality of life |
| ACER | Average cost-effectiveness ratio |
| BMI | Body mass index |
| BMS | Breastmilk substitutes |
| BSID-III | Bayley Scales of Infant and Toddler Development – III |
| CBA | Cost-benefit analysis |
| CCA | Cost-consequence analysis |
| CEA | Cost-effectiveness analysis |
| CEAC | Cost-effectiveness acceptability curve |
| CHC | Child health care centre |
| CHEERS | consolidated health economic evaluation reporting standards |
| CUA | Cost-utility analysis |
| DALY | Disability-adjusted life year |
| ECD | Early childhood development |
| EQ-5D | The European Quality of Life 5 Dimensions |
| GDM | Gestational diabetes mellitus |
| HEAP | Health economic analysis plan |
| HRQoL | Health related quality of life |
| HSUV | Health state utility value |
| ICER | Incremental cost-effectiveness ratio |
| NCD | Non-communicable disease |
| NHS | National Health Service |
| PA | Physical activity |
| PICO | Population, intervention, comparator, outcome |
| PRISMA | Preferred Reporting Items for Systematic reviews and Meta-Analyses |
| PSA | Probabilistic sensitivity analysis |
| QALY | Quality adjusted life year |
| RCT | Randomised controlled study |
| RVI | Relative value index |
| SF-36 | 36-Item Short Form Health Survey |
| SF-6D | Short-Form 6-Dimension |
| VAS | Visual analogue scale |
| WIC | Women, infants, children |
| WTP | willingness-to-pay |
